# Mitophagy: An Emergence of New Player in Alzheimer’s Disease

**DOI:** 10.3389/fnmol.2022.921908

**Published:** 2022-07-06

**Authors:** Bunty Sharma, Deeksha Pal, Ujjawal Sharma, Aman Kumar

**Affiliations:** ^1^Department of Biotechnology, Maharishi Markandeshwar (Deemed to be University), Mullana, Haryana, India; ^2^Department of Nephrology, Postgraduate Institute of Medical Education and Research, Chandigarh, India; ^3^Department of Ophthalmology and Visual Sciences, Ohio State University, Columbus, OH, United States

**Keywords:** mitophagy, Alzheimer’s disease, mitochondrial quality control, mitochondrial dynamics, mitochondrial dysfunction, targeting mitophagy

## Abstract

Mitochondria provide neurons not only energy as ATP to keep them growing, proliferating and developing, but they also control apoptosis. Due to their high bioenergetic demand, neurons which are highly specific terminally differentiated cells, essentially depend on mitochondria. Defective mitochondrial function is thus related to numerous age-linked neurodegenerative ailments like Alzheimer’s disease (AD), in which the build-up of impaired and malfunctioning mitochondria has been identified as a primary sign, paying to disease development. Mitophagy, selective autophagy, is a key mitochondrial quality control system that helps neurons to stay healthy and functional by removing undesired and damaged mitochondria. Dysfunctional mitochondria and dysregulated mitophagy have been closely associated with the onset of ADs. Various proteins associated with mitophagy were found to be altered in AD. Therapeutic strategies focusing on the restoration of mitophagy capabilities could be utilized to strike the development of AD pathogenesis. We summarize the mechanism and role of mitophagy in the onset and advancement of AD, in the quality control mechanism of mitochondria, the consequences of dysfunctional mitophagy in AD, and potential therapeutic approaches involving mitophagy modulation in AD. To develop new therapeutic methods, a better knowledge of the function of mitophagy in the pathophysiology of AD is required.

## Introduction

Alzheimer’s disease (AD) is a progressive neurodegenerative disorder that affects brain cells to shrink and gradually die (Cai and Jeong, 2020). Dr. Alois Alzheimer, a German physician, identified a memory loss condition along with microscopic changes in the brain. Later Emil Kraepelin named the condition AD. Memory loss is an initial symptom of AD, as the disease gets worsens; it leads to loss of cognitive function causing a person’s inability to perform daily activities independently. Common symptoms of ADs are depression, delusion, irritability, sleeplessness and social withdrawal (Revi, 2020).

There are several factors implicated in AD development and progression. Defective mitochondrial clearance is one of the key factors in AD occurrence, as well as severity (Pradeepkiran and Reddy, 2020). Mitochondria are double membranous organelles in the eukaryotic cell, known as the powerhouse of the cell. Energy is generated in the form of ATP (adenosine triphosphates) molecules by the aerobic respiration process. Impaired mitochondrial function has been implicated in AD, Lou Gehrig’s disease, Muscular dystrophy, Diabetes, Cancer, Huntington’s disease, and Parkinson’s disease (Nunnari and Suomalainen, 2012). Mitochondria are under strict quality control dynamics in the cell. Defective mitochondria are replaced by an autophagy cell death mechanism termed mitophagy.

The basic event in AD is the formation and accumulation of Amyloid-β that induces hyperphosphorylation of tau proteins. Amyloid-β and phosphorylated tau (p-tau) proteins abnormally interact with mitochondrial functional proteins such as DRP1 (Dynamin-related protein 1) and PINK1 (PTEN-induced putative kinase protein 1). This abnormal interaction ends up in deficient mitochondrial clearance or mitophagy (Reddy and Oliver, 2019). Pilling up of damaged mitochondria causes energy deficiency, the release of reactive species, oxidative stress and impedes signaling cascade which ultimately leads to neurodegenerative disorders.

Mitophagy, as well as mitochondrial dynamics, are being explored as a novel therapeutic avenue in AD treatment. In this regard, there are several small molecule inhibitors are being tested and have shown promising effects in modulating the function of various proteins involved in AD development and progression. One such molecule is DRP1 whose inhibitors i.e., Diethyl(3,4-dihydroxyphenethylamino) (quinolin-4-yl) methyl phosphonate (DDQ), mitochondrial division inhibitor 1 (Mdivi-1) and Dynasore have shown efficacy in preclinical studies in AD (Kandimalla et al., 2021; Medala et al., 2021).

Mitochondrial impairment is central to AD’s development. In the past few years, immense data have been generated showcasing dysfunctional mitophagy as a mediator for AD. Therefore, it is extremely important to understand mitophagy dysfunction and molecules involved in the process eventually leading to AD development and progression. Factoring in the relevance of mitophagy in AD, we have made an attempt to summarize the different aspects of mitophagy in AD and its prospects as therapeutics. We have discussed recent advancements in the identification of molecular targets involved in impaired mitophagy as well ADs. The review will provide the readers with a comprehensive overview of mitophagy mechanistic, regulators, triggers and its scope as therapeutics.

### Mitophagy: Mechanism and Regulation

Mitophagy is an evolutionarily conserved mechanism for the replacement of damaged mitochondria and safeguarding cells from aberrant cell death signaling. Mitophagy consists of four basic steps (i) Initiation of mitophagic process. (ii) Preparation of mitochondria for mitophagy process to be identified by autophagic machinery. (iii) Mitochondrial engulfment & formation of mitophagosome. (iv) Lysosomal degradation.

During stress, DRP1 gets localized at the mitochondrial-associated endoplasmic reticulum membrane and inhibition of mitochondrial fusion is controlled by mitofusin 1 & 2 (Mfn1 & 2) and optic atrophy 1 (OPA1) initiates mitophagy. In damaged mitochondria, PINK1 degradation by presenilin-associated rhomboid-like protease and matrix protein mitochondrial processing peptidase is inhibited hence stabilizing PINK1 present on the outer mitochondrial membrane (OMM). PINK1 phosphorylates both ubiquitin and parkin, further they ubiquitinate several OMM proteins signals recognition of mitochondria by autophagic machinery and initiate mitophagosome formation through binding to microtubule-associated protein 1 light chain 3 (LC3). Next, the fusion of mitophagosome with lysosome generates mitolysosome and leads to the elimination of faulty mitochondria (Zuo et al., 2020).

**FIGURE 1:**
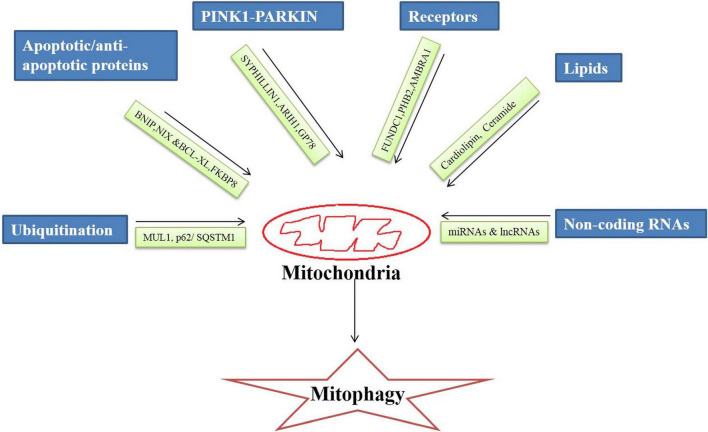
Regulators of mitophagy process.

Broadly, the mitophagy mechanism is of two types ubiquitin-dependent and ubiquitin-independent. It is regulated by an intricate network of molecules that on different stimuli by the cellular environment execute the mitophagy process. There are specialized regulators molecules involved in the mitophagy mechanism that are discussed below (Figure 1).

### Ubiquitin

Ubiquitins are small molecular weight (8.5 KDa) proteins involved in the proteasomal degradation of proteins (Garcia-Barcena et al., 2020). Ubiquitins bind to Lys, Cys, Cys/Thr, or Ser/Thr residues at the protein substrates, form the isopeptide linkage and initiates ubiquitin-dependent mitophagy. Ubiquitin E3 ligase enzymes catalyze the transfer of ubiquitin to the protein substrate initiating the autophagic process (Zheng and Shabek, 2017).

In the cell, there are hundreds of different types of ubiquitin E3ligases which under the different stimuli of cellular signaling process different protein substrates. Mitochondrial ubiquitin ligase 1 (MUL1) or mitochondrial-anchored protein ligase (MAPL1) or mitochondrial ubiquitin ligase activator of NF-κB (MULAN) are the key players in the mitochondrial degradation of proteins. MULI, OMM protein, ring finger domains face toward the cytoplasm. MUL1 interacts with four E2-conjugating enzymes (Ube2E2, Ube2E3, Ube2L3, and Ube2G2) during ubiquitination and signals mitochondrial clearance (Ambivero et al., 2014).

p62/SQSTM1 (sequestosome 1) is a ubiquitin-binding scaffold that linked the mitochondrial ubiquitinated proteins to the mitophagy process by binding to LC3 simultaneously. p62/SQSTM1 regulates polyubiquitination of OMM proteins *via* the keap1-Nrf2 pathway.

### PINK1-PARKIN Pathway

PINK1-PARKIN dependent is the most common and well-studied pathway of mitophagy (Iorio et al., 2021). Loss of mitochondrial potential causes accumulation of PINK1 at the damaged mitochondria which signals translocation of PARKIN, an E3 ubiquitin ligase at the damaged membrane of mitochondria. PARKIN initiates the ubiquitination of proteins of the OMM and the clearance of damaged mitochondria *via* the autophagic process (Eiyama and Okamoto, 2015).

It has been observed that in drosophila mutated PINK1 and PARKIN proteins lead to a significant decrease in mitochondrial protein turnover as well as activation of the alternative pathway of protein turnover pointing to the significance of the PINK1-PARKIN pathway in mitophagy. Mutations in PINK1 and PARKIN have been implicated in neurogenerative disorders like AD (Doblado et al., 2021). The Smad ubiquitin regulatory factor 1 (SMURF1), an E3 ubiquitin ligase is essential for mitophagy. It ubiquitinates several proteins for proteasomal degradation (Franco et al., 2017). In a PARKIN independent pathway PINK1 recruits SYNPHILIN1, recruitment of SYNPHILIN1 is independent of the kinase activity of PINK1, which in turn recruits SIAH1 (seven *in absentia* homolog 1). It recruits E3 ubiquitin ligase that leads to ubiquitination and proteasomal degradation of mitochondrial proteins (Szargel et al., 2016).

Another, E3 Ubiquitin protein ligase 1 Ariadne (ARIH1) belongs to RING-between-RING E3 ligases and induces mitophagy in PINK1 dependent and PARKIN independent manner (Yao et al., 2021). ARIH1 (Molecular weight 64 KDa) has structural and functional similarities with PARKIN. PINK1 phosphorylates serine and threonine residues in the aridane domain of ARIH1 thus unmasking the RING type 2 domain containing catalytic site. Phosphorylation of ARIH1 by PINK1 is the initial and critical step of ARIH1-mediated mitophagy (Villa et al., 2017).

Glycoprotein (GP78) is also an E3 ubiquitin ligase that mediates mitophagy independent of PARKIN. GP78 is located in ER domain associated with mitochondria. A study has revealed that Mfn1 & Mfn2 undergo ubiquitination catalyzed by GP78 leading to mitochondrial fragmentation (Fu et al., 2013).

### Apoptotic Proteins

Proapoptotic proteins are involved in mitochondrial protein degradation and recycling irrespective of ubiquitination. BCL2L13 (BCL2 like 13), BNIP [NIP3 (BCL2/adenovirus E1B 19 KDa protein-interacting protein 3) and NIX (NIP3-like protein X)/BNIP3L, are located on OMM induce mitochondrial fragmentation and recruit autophagic machinery through their interaction with LC3 protein *via* their LIR (LC3 interacting) motif (Novak et al., 2010). BNIP and NIX proteins trigger mitophagy in response to hypoxia conditions (Zhang and Ney, 2009; Weckmann et al., 2018). In the MALM (Mieap-induced accumulation of lysosome-like organelles within mitochondria) pathway, BNIP3 or BNIP3L localized on OMM interacts with Mieap (mitochondria-eating protein) leading to pore formation in the mitochondrial membrane thus enabling lysosomes to enter into the mitochondria (Kamino et al., 2016). MALM pathway is a recently discovered pathway to maintain mitochondrial quality in the cells by removing unhealthy mitochondria (Okuyama et al., 2019). Whereas BCL2L13 is involved in starvation-induced mitophagy through interaction with the ULK1 complex (Ney, 2015).

In contrast, B-cell lymphoma-extra-large (Bcl-xL), a transmembrane anti-apoptotic protein localized on the mitochondria, inhibits mitophagy induction by suppressing PARKIN translocation to the depolarized mitochondrial membrane or by binding to PINK1 thus inhibiting the signal relay to PARKIN (Gross and Katz, 2017). On the contrary FKBP8 (FK506-binding protein 8), which is also an anti-apoptotic protein induces stress-induced mitophagy independent of PARKIN. FKBP8 induces mitophagy by binding to LC3A and recruiting autophagosomes to mitochondria under stress conditions (Shirane-Kitsuji and Nakayama, 2014).

### Receptors

FUN14 domain containing 1 (FUNDC1), is an OMM receptor playing role in mitophagy induction in hypoxia as well as stress conditions. It also carries out mitophagy by interacting with LC3 through its LIR domain (Liu et al., 2019). There is also one receptor localized on the inner mitochondrial membrane that activates mitophagy is PHB2 (Prohibitin 2), PHB2 along with PHB/PHBI forms prohibit complexity at the inner mitochondrial membrane and induce PARKIN dependent mitophagy. PHB2 interacts with LC3II through its LIR domain and causes mitochondrial clearance (Yan et al., 2020). Also, AMBRA1 (Activating Molecule in BECLIN1-Regulated Autophagy1) an inner receptor can induce PARKIN or p62 independent mitophagy. It can directly interact with LC3 *via* its LIR domain located at the c-terminal domain and induce mitophagy (Strappazzon et al., 2015).

### Lipids

Lipids are integral components of cellular membranes. During mitochondrial stress or damage, the lipids can activate the autophagic process for damaged mitochondrial clearance. Cardiolipin (CL) is a lipid moiety present in the inner membrane of mitochondria that flips outside during mitophagy and binds with the preferentially LC3A component thus initiating mitophagy. Phospholipid scramblase-3(PLSCR3) facilitates the externalization of CL to the outer membrane. Also, CL has an affinity to Beclin1 protein, a key protein in autophagy thus initiating the mitophagy process (Schlame and Greenberg, 2017).

Ceramide is a sphingolipid, a key component in inducing lethal mitophagy. Stress signaling activates DRP1 which elicits mitochondrial fission followed by mitochondrial damage. Further, there is the translocation of ceramide synthase 1 protein to OMM which in turn generate the C18-ceramide that binds with LC3B and results in mitophagy (Vos et al., 2021).

### Non-coding RNAs

MicroRNAs are single-stranded non-coding RNAs that are 22 nucleotides long and participate in the post-transcriptional regulation of genes (O’Brien et al., 2018). Data related to the role of miRNAs in AD *via* mitophagy regulation is primitive. Approximately 400 mitochondrial miRNAs (mito-miRNAs) are being identified as regulating the mitochondrial genome. Most recently published literature has confirmed the role of mito-miRNAs in mitophagy regulation.

Long non –coding RNAs (lncRNAs) are 200 long nucleotide transcripts that do not translate into proteins. A lncRNAs H19’s role has been implicated in mitophagy regulation. A study by Wang et al. (2021), have shown that PINK1, a canonical pathway involved in mitophagy is inhibited by H19 by restricting PINK1 mRNA translation. Another study has revealed that lncRNA SNHG14 (small nucleolar RNA host gene 14), activates mitophagy by upregulating the expression of BNIP3, Beclin-1 and LC3II/LC3I ratio (Deng et al., 2020). Also, the findings of a recent study uncovered that the lncRNA NEAT1(nuclear paraspeckle assembly transcript 1)- miR-150-5p-DRP1 axis regulates mitophagy (Yang et al., 2021). There are several studies published in recent years confirming epigenetic regulation of mitophagy.

### Mitochondrial Quality Control Mechanisms and Cellular Homeostasis in Alzheimer’s Disease

Mitochondria, the powerhouse of the cell, is not only accountable for the production of ATP as an energy source but also a major player in the intrinsic cell death pathway activation that leads to cell death, making it a key constituent of the eukaryotic cell. Besides energy production, mitochondria participate in several other processes for example neuronal ischemia-reperfusion, innate immunity and aging (Osellame and Duchen, 2014). Henceforth, faulty mitochondria will almost likely disrupt cell and tissue functioning and jeopardize the health of the entire organism. Therefore, it is critical to maintain viable populations of mitochondria for normal cellular function.

Mitophagy, a pathway for the disposal of defective mitochondria, performs a crucial role in conserving the integrity as well as the quality of mitochondria. Mitophagy is considered a protective cellular process by reducing reactive oxygen species production. The malfunctioning of mitochondria and defective mitophagy may lead to aging and various neurodegenerative disorders like AD.

The system of mitochondrial quality control (mitoQC) overawed the deformities in mitochondria, including biogenesis and dynamics of mitochondrial mitophagy and proteostasis (Anzell et al., 2018). Therefore, mitoQC denotes an overall equilibrium between mitochondrial formation and mitochondrial degradation. The morphology and volume of mitochondria are maintained by the homeostasis among dynamics, fission, and fusion of mitochondria. Mitochondrial biogenetic failure is a critical activator of inflammatory response pathways, indicated by an increase in oxidative stress, cytoplasmic calcium and reduction of mtDNA (Yan et al., 2020). Various mechanisms, particularly the Parkin/PINK1 pathway, are used to eliminate defective mitochondria (Whitworth and Pallanck, 2017).

Several studies have shown that in AD, there is a disproportion between fusion and fission of mitochondria, which contributes to the disease pathogenesis. The altered differentially protein expression, involved in the fusion and fission of mitochondria, was observed in the hippocampus region of the brain of an AD patient. This involved elevated expression of Fis1 and a decline in DRP1 expression, along with fusion proteins like OPA1, Mfn1 and Mfn2 expression (Chakravorty et al., 2019). Furthermore, DRP1 phosphorylation and S-nitrosylation were higher in the brains of AD patients than in controls, proving the activation of extreme mitochondrial fission (Wang et al., 2009). The levels of mRNA expression of genes for mitochondrial fission-related proteins e.g., Fis1 are also raised in the AD patient’s blood (Pakpian et al., 2020). The accumulation of tau protein in pluripotent stem cells (iPSCs) induced neurons from AD patients is linked to augmented expression of proteins related to mitochondrial fission (Lee et al., 2018). Thus, extreme fission combined with the declined fusion of mitochondria might result in bioenergetic inefficiency, which can be involved in the development of AD.

Multiple cell cultures and animal models of AD, as well as brains from AD patients after post-mortem, have shown the irregular distribution of mitochondrial and neuronal transport (Wang et al., 2017). One of the common axon pathologies in AD is axonal transport dysfunction and both Tau and Aβ can lead to disruption of axonal mitochondrial transport (Zheng et al., 2019). Mitochondrial trafficking along the axon is mediated by the mitochondrial protein Miro. The mutation in the miro gene has been formerly found to be linked with the Aβ42 distribution in the Drosophila model of AD (Yan et al., 2020). The metabolic stress seen in AD is linked to an abnormal mitochondrial permeability transition pore (MtPTP) protein. Downregulation of OPA1 altered mitochondrial dynamics, resulting in expansion of mitochondria and impaired function of MtPTP (Yan et al., 2020). Furthermore, syntaphilin, an anchor protein of mitochondria, is degraded in human APP-expressing neurons related to AD, resulting in mitochondrial rearward trafficking (Hu et al., 2021). These findings imply that dynamics and transport of mitochondria are disrupted in AD, potentially leading to synaptic dysfunction and neurodegeneration.

Overall, this implies that impaired mitochondrial QC may play a role in the pathogenesis of AD and that therapeutically addressing these pathways could be a reasonable way of treating the disease.

### Impaired Mitophagy in Alzheimer’s Disease: Metabolic and Molecular Triggers

The disturbances in mitophagy are a common feature in many neurodegenerative diseases including AD, resulting in reduced capacity to eliminate dysfunctional mitochondria (Kerr et al., 2017). It has been seen that mitophagy is even less than 50% in AD patients when compared to healthy controls and inhibition of mitophagy can lead to the accumulation of dysfunctional neurons in AD (Lou et al., 2020). The fusion of mitochondria containing autophagosome with lysosome is a critical step in the process of mitophagy and abnormal accumulation of autophagosomal vacuoles in the neuronal cell bodies is often observed in AD (Nixon, 2013). Serval regulatory proteins of mitophagy are found to be alerted in AD patients such as presenilin 1 (PS1), phosphatase and PINK1, Bcl-2 associated athanogene 3 (BAG-3), p62, Microtubule-associated protein 1A/1B-LC3 and TANK-binding kinase 1 (TBK1; Xie et al., 2022). The levels of PARKIN, which is E3 ubiquitin ligase and required to initiate the mitophagy, were found to be reduced in the cytoplasm of AD patient brains along with abnormal PINK1 accumulation, leading the defective mitophagy, which in turn can be resorted by overexpressing PARKIN (Martín-Maestro et al., 2016). Additionally, the ataxia telangiectasia mutated is also found to be downregulated in AD, which is a DNA repair gene, known to play an important role during the process of mitophagy (Amirifar et al., 2019). The lysosomal activity is one of the critical factors for mitophagy and any defect in the proteolysis capacity of lysosomes can impair mitophagy. The inhibition of lysosomal proteolysis activity in wild-type mice was shown to imitate the neuropathology of AD (Lee et al., 2011). In addition, the genetic mutations in PS1 affecting the activity of lysosomal hydrolase can result in disturbances in mitophagy (Lee et al., 2010; Coffey et al., 2014). Another important mutated gene is mitochondrial rho (MIRO), necessary for the activation of PINK1 and PARKIN during the mitophagy process (Saito and Sadoshima, 2015). The reduction in PINK1 and PARKIN in AD is the most powerful event for AD development as it increases the number of non-functional mitochondria due to defective mitophagy. These damaged mitochondria in turn lead to oxidative stress and inflammation through reactive oxygen species (ROS) and interleukin production (Fang et al., 2019; Oliver and Reddy, 2019). Accumulation of 99-aa C-terminal fragment of amyloid precursor protein (APP-C99) also leads to increased ROS levels and mitophagy suppression by disturbing the cristae organization (Vaillant-Beuchot et al., 2021). The expression of mitochondrial biogenesis genes such as peroxisome proliferator-activated receptor-y coactivator 1α (PGC-1α), transcription factor A and mitochondrial and nuclear factor NRF2 are also altered in AD patients (Rice et al., 2014). The other important regulators of mitophagy are sirtuins (SIRT) which are nicotinamide adenine dinucleotide (NAD+) dependent histone deacetylases and play an essential role in transcription regulation and cellular metabolism. The SIRT family comprises seven members ranging from SIRT1-SIRT7, and only SIRT3, SIRT4 and SIRT5 are exclusively found in the mitochondria. Studies in AD patients found decreased expression of SIRT1, which is mainly nuclear protein but shuttles between the nucleus and cytoplasm. SIRT1 induces mitophagy by activating the PGC-1α and other autophagy proteins (Julien et al., 2009; Fang et al., 2014). Similarly, SIRT3 levels are decreased in AD, which also activates mitophagy by inducing p62 levels. SIRT3 also protects mitochondria against metabolic stress by activating superoxide dismutase 2 and cyclophilin D deacetylation-dependent mechanism (Tseng et al., 2013; Yang et al., 2015; Cheng et al., 2016). Recently, Baeken et al. (2021) showed that SIRTs are themselves targets of autophagic degradation in the neuronal cells under oxidative stress in Parkinson’s disease models. However, the activity and degradation of SIRTs are regulated by independent mechanisms therefore, more experimental data are needed to delineate the homeostasis of SIRTs in mitophagy under oxidative stress conditions in AD (Beaken et al., 2021). As SIRTs are dependent on NAD, the levels of both oxidized (NAD+) and reduced (NADH) forms of NAD are important for mitophagy. Reduced NAD levels are found to exacerbate AD pathology by altering mitophagy (Zhou et al., 2015). Furthermore, enzymes other than SIRTs that use NAD as a co-factor are found to be alerted in neurodegenerative diseases such as poly (ADP-ribose) polymerase 1 (PARP1), and cyclic ADP ribose hydrolase (CD38) (Verdin, 2015). PARP1 is a DNA damage response enzyme, involved in poly ADP-ribosylation (PARylation) of target proteins using NAD+ while CD38 is a surface glycoprotein, that catalyzes the production of cyclic adenosine diphosphate ribose, and ADP ribose using NAD+. The levels of both PARP1 and CD38 were found to be higher in AD as compared to controls, which reduces the NAD+ levels and thereby decreases the SIRT activity (Strosznajder et al., 2012; Fang et al., 2016; Martire et al., 2016). The tau protein is also linked to suppress mitophagy by disturbing the mitochondrial membrane potential and by reducing levels of PARKIN as shown by Hu et al. (2016). Tau was also observed to be physically interacted with PARKIN leading to cytosolic sequestration of protein and thereby inhibiting its translocation to mitochondria (Cummins et al., 2019). Mitochondrial dynamics are usually balanced by two types of proteins known as mitochondrial fission proteins (Fis1) and DRP1 and fusion proteins (Opa1, Mfn1, and Mfn2). The role of the fission proteins is the degradation of misfolded mitochondrial proteins while fusion proteins allow the attachment of mitochondria to the intracellular structures and help to dilute the damaged content (Burté et al., 2015). Manczak et al. (2011) have reported the interactions between the Aβ and mitochondrial fission protein DRP1, which increases with the disease progression, demonstrating the important role of Aβ protein in mitophagy. They also found decreased levels of Mfn2 as well as mitophagy in AD patients. In addition, phosphorylated tau (p-tau) was also found to be associated with DRP1 protein and further leads to excessive fragmentation of mitochondria in AD (Manczak and Reddy, 2012).

Therefore, the balance between the mitochondrial biogenesis and degradation are critical for maintaining the mitochondrial quality control, and any disturbance of which can lead to the accumulation of dysfunctional mitochondria and worsens the AD pathology. Till now, the studies have suggested the importance of reduced mitophagy in the maintenance of AD homeostasis. Therefore, the interventions that can stimulate the mitophagy process might be helpful in improving the cognitive functions in AD patients.

### Targeting Mitophagy: An Approach to Treat Alzheimer’s Disease

The mitoQC ensures that the functions of mitochondria are maintained. The mitoQC components that control mitochondrial dynamics and mitophagy are having considerably changed expression in AD patients and experimental systems. Mitophagy can be restored with an increase in levels of proteins regulating mitophagy in both *in-vitro* and *in-vivo* AD models.

Recently, the pharmacological regulation of mitophagy was found to improve pathology related to Aβ and tau, along with the cognitive deficits related to it, in several AD models. Urolithin A (UA), nicotinamide mononucleotide (NMN), and actinonin (AC), three powerful mitophagy inducers discovered in a screen, reversed the pathogenesis of AD (Fang et al., 2019).

In neurons and muscles, UA which is polyphenol ellagitannins metabolite has been shown to cause mitophagy. UA treatment-induced mitophagy in SH-SY5Y cells (human neuroblastoma cell lines) by increasing the expression levels of parkin, p-ULK1, PINK1, AMBRA1, BECN1, and Bcl2L13, among other mitophagy-related proteins. Parkin overexpression can improve functions of mitochondria, reinstate PINK levels, boost the generation of ATP, and reduce levels of ubiquitinated Aβ in APP/PS1 animals and cells treated with Aβ (Wang H. et al., 2020). UA therapy lowered overall Aβ levels and improved memory in an AD model of *C. elegans* with Aβ42 expression in all neurons. Pink-1 and pdr-1, two important mitophagy genes, were required for this increase in cognitive function. UA’s preventive role in AD *via* mitophagy appears to be preserved across animals. With UA therapy, a transgenic mice model displayed enhanced learning and improved memory, as well as lower expression of amyloid peptides Aβ40, Aβ42, and Aβ plaques of extracellular origin (Fang et al., 2019). Both AD models (*C. elegans* and mice) showed improved AD pathogenesis after UA therapy by suppressing tau phosphorylation in a mitophagy-dependent way.

The precursors of NAD + like nicotinamide riboside have shown the improvement in pathological characteristics in AD models. In an AD mouse model, nicotinamide riboside therapy reduced phosphorylation of tau and enhanced synaptic and cognitive function, which was demonstrated to be facilitated by induction of mitophagy (Hou et al., 2018, 2019). The induction of mitophagy by NAD + precursors could be a favorable therapeutic strategy for AD treatment because NAD + works as a cofactor for several proteins that regulate the autophagy/mitophagy pathway, including SIRT, PARP (poly [ADP-ribose] polymerase) and SARM1 (sterile alpha and TIR motif-containing 1) (Fang et al., 2019). Furthermore, nicotinamide riboside (NR) enhances mitophagy by increasing levels of proteins related to mitophagy e.g., LC3 (Aman et al., 2020). Further, S14, a phosphodiesterase (PDE)-7 inhibitor has shown neuroprotection in APP/PS1 mice *via* moderating mitochondrial dysfunction induced by Aβ *via* restoring expression levels of LC3 (Bartolome et al., 2018).

AC, a natural antibacterial drug, was likewise found to cause mitophagy in neurons and affects AD pathogenesis similar to UA therapy. The essential mitophagy genes PINK-1, DCT-1, and PDR-1 were required for mitophagy activation in an AD model of *C. elegans*. AC increased cognitive capacity and mitochondrial functions in AD models, as well as reduced Aβ plaque load (Fang et al., 2019). Furthermore, in iPSC-derived neural stem cells mutant (PS1) related to AD, bexarotene re-established mitophagy and repaired the altered topology of the mitochondrial network (Martin-Maestro et al., 2019).

Mitophagy activity can also be influenced by enzymes such as SIRT and adenylate-activated protein kinase (AMPK). In streptozotocin (STZ) mice, for example, AMPK overexpression lowers the phosphorylation of tau (Wang L. et al., 2020). Furthermore, activators of SIRT like resveratrol promote mitophagy *via* the mTOR-ULK1 (Unc-51 Like Autophagy Activating Kinase 1) pathway (Park et al., 2020). SIRT activators have shown to be proven in clinical trials to affect levels of Aβ and markers of inflammation in AD patients. Mitophagy appears to participate in reducing inflammation, and thus manages inflammation of neurons in AD. The PINK1 and parkin, proteins related to mitophagy, were found to help attenuate STING-induced inflammation (Sliter et al., 2018). The mitophagy induction in microglia lowered inflammation and AD development (Fang et al., 2019). Because of their prolonged activated state, microglia produce more pro-inflammatory cytokines including interleukin-6 and tumor necrosis factor-α and while producing less anti-inflammatory cytokine interleukin -10 as AD progresses (Lautrup et al., 2019).

The elicitation of mitophagy is linked with reduced NLRP3 inflammasome activation, as well as the lesser proportion of its target molecules like activated caspase-1 and IL-1β in the mouse model of AD. All these observations, together with the decreased insoluble Aβ plaques, suggest that pharmacologically restoring mitophagy improves phagocytosis and reduces NLRP3- mediated inflammation in AD models, therefore improving AD pathogenesis (Fang et al., 2019; Lautrup et al., 2019). Moreover, the inducer of autophagosomes *via* lysosomes, such as trehalose, can also stimulate mitophagy in AD (Tien et al., 2016).

As a result, modulating mitophagy in AD may protect both microglia and neurons, highlighting it as a plausible therapeutic target for AD.

## Conclusion and Future Direction

The maintenance of mitochondrial integrity is an important aspect of a cell’s ability to operate properly. According to new findings, defects in MQC may lead to the progression of AD pathogenesis. The anomalous function of mitochondrial, deprived mitochondrial dynamics and imbalanced mitophagy have been linked with synaptic dysfunction, increased oxidative stress, loss of neurons and declined cognitive ability in several AD models across species, contributing to exacerbated AD pathogenesis. Our understanding of the molecular mechanisms driving mitophagy has vastly improved during the last decade. Because of the importance of mitophagy in the course of AD, pharmacological approaches that target these pathways have been tested in many AD models. Mitophagy is responsible for removing damaged mitochondria. As a result, activation of mitophagy may help to replenish the pool of healthy mitochondria in AD, where stressed and damaged mitochondria have accumulated.

Mitophagy activation has been found to be protecting the pathogenesis of AD by lowering the accumulation of Aβ plaque and inflammation in neurons, hence postponing cognitive loss. Despite mounting evidence of the therapeutic benefits of targeting mitophagy in AD, any significant differences between experimental models and human participants should be carefully acknowledged.

The adverse effects of treatment techniques aimed at upregulating mitophagy on the mitochondrial function within the healthy pool must be assessed. As a result, a greater knowledge of the link between mitophagy and AD pathogenesis could lead to the development of effective and safe treatments for the disease. Despite the fact that great progress has been made in this field, further studies are needed to create and validate more effective mitophagy inducers that could eventually be used as a therapeutic technique to combat the pathogenesis of AD.

## Author Contributions

BS and DP: literature review, manuscript writing, and final approval of the manuscript. US: conception and design, manuscript writing, and final approval of the manuscript. AK: manuscript writing and final approval of the manuscript. All authors contributed to the article and approved the submitted version.

## Conflict of Interest

The authors declare that the research was conducted in the absence of any commercial or financial relationships that could be construed as a potential conflict of interest. The reviewer SK declared a shared affiliation with one of the author DP to the handling editor at the time of review.

## Publisher’s Note

All claims expressed in this article are solely those of the authors and do not necessarily represent those of their affiliated organizations, or those of the publisher, the editors and the reviewers. Any product that may be evaluated in this article, or claim that may be made by its manufacturer, is not guaranteed or endorsed by the publisher.
